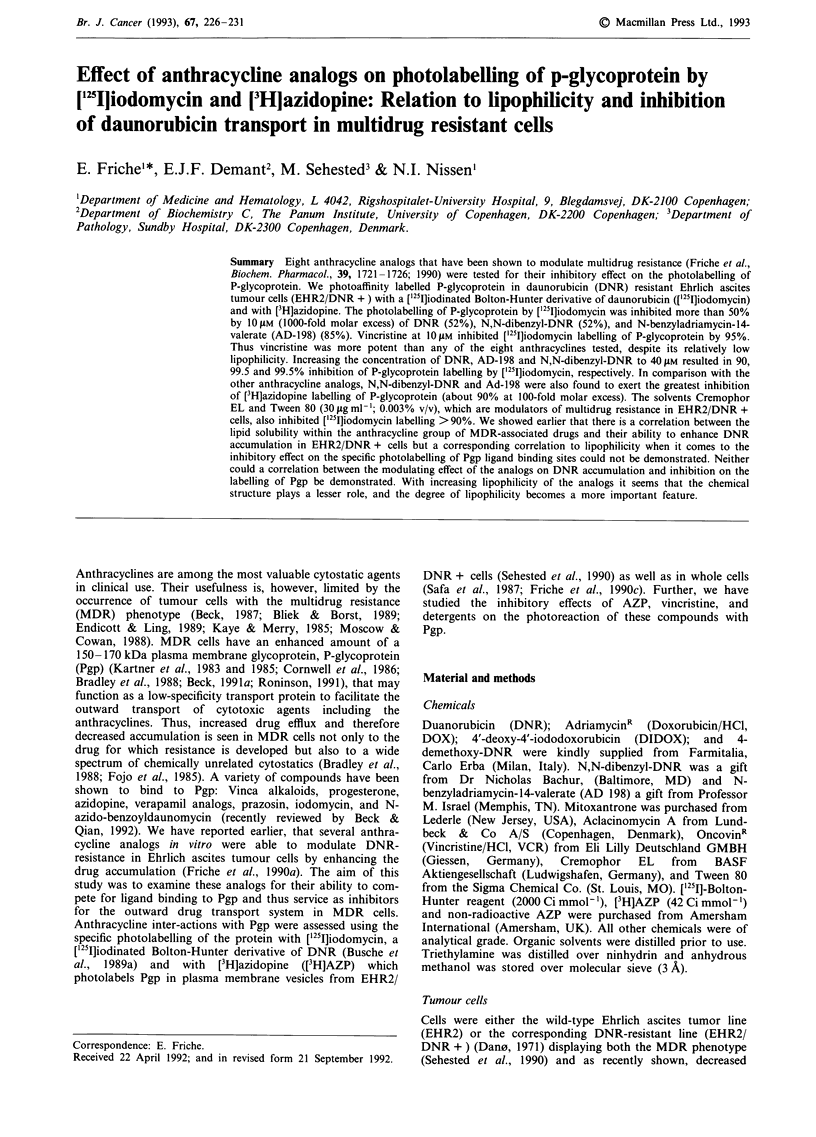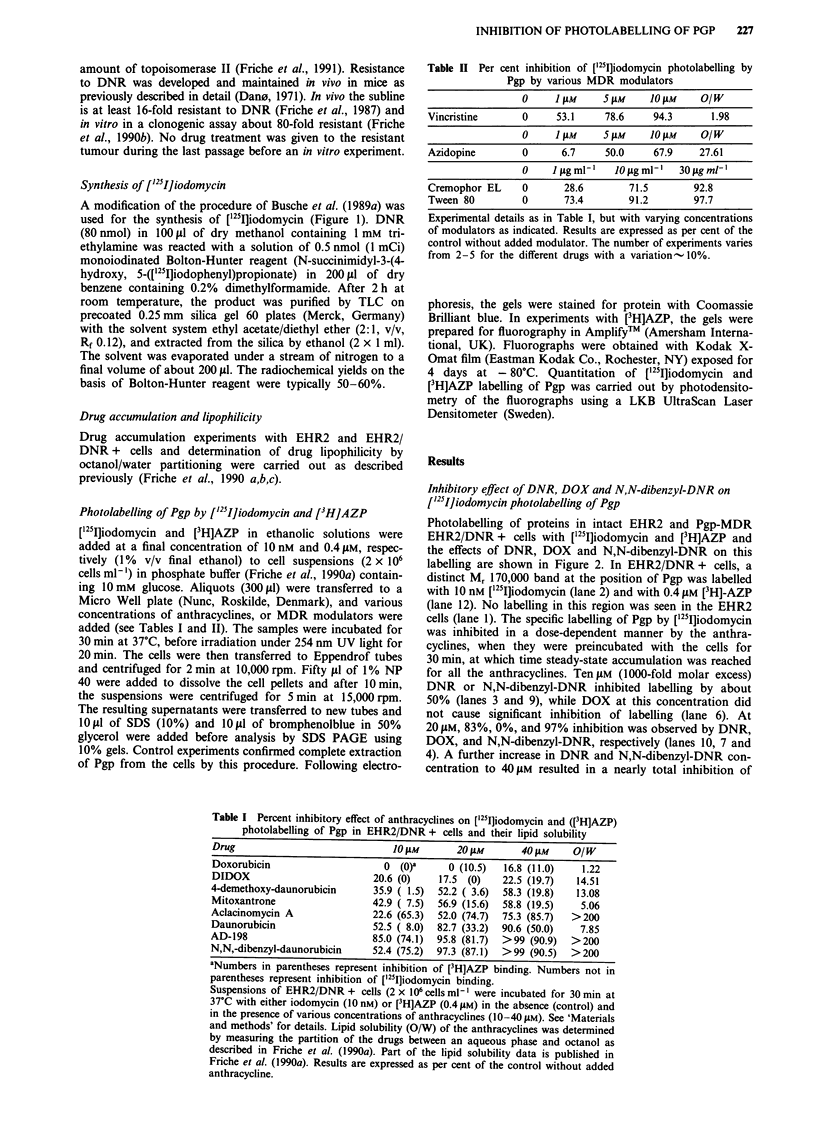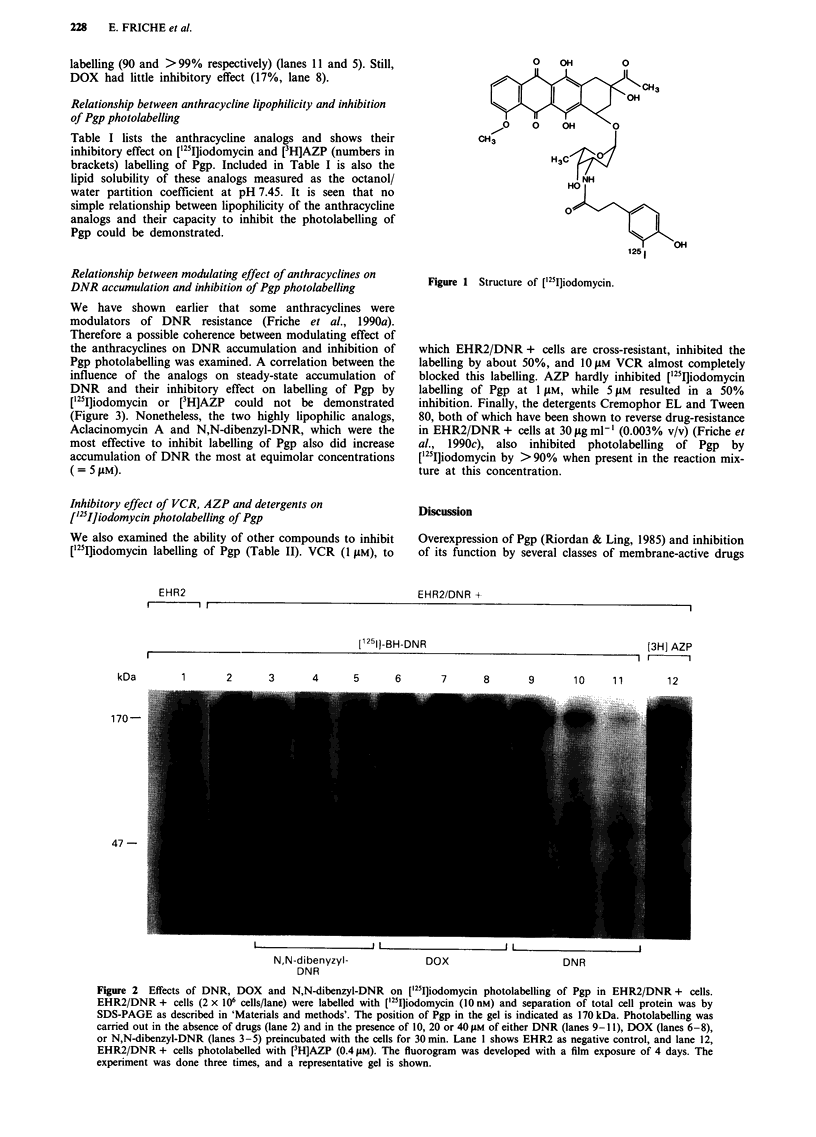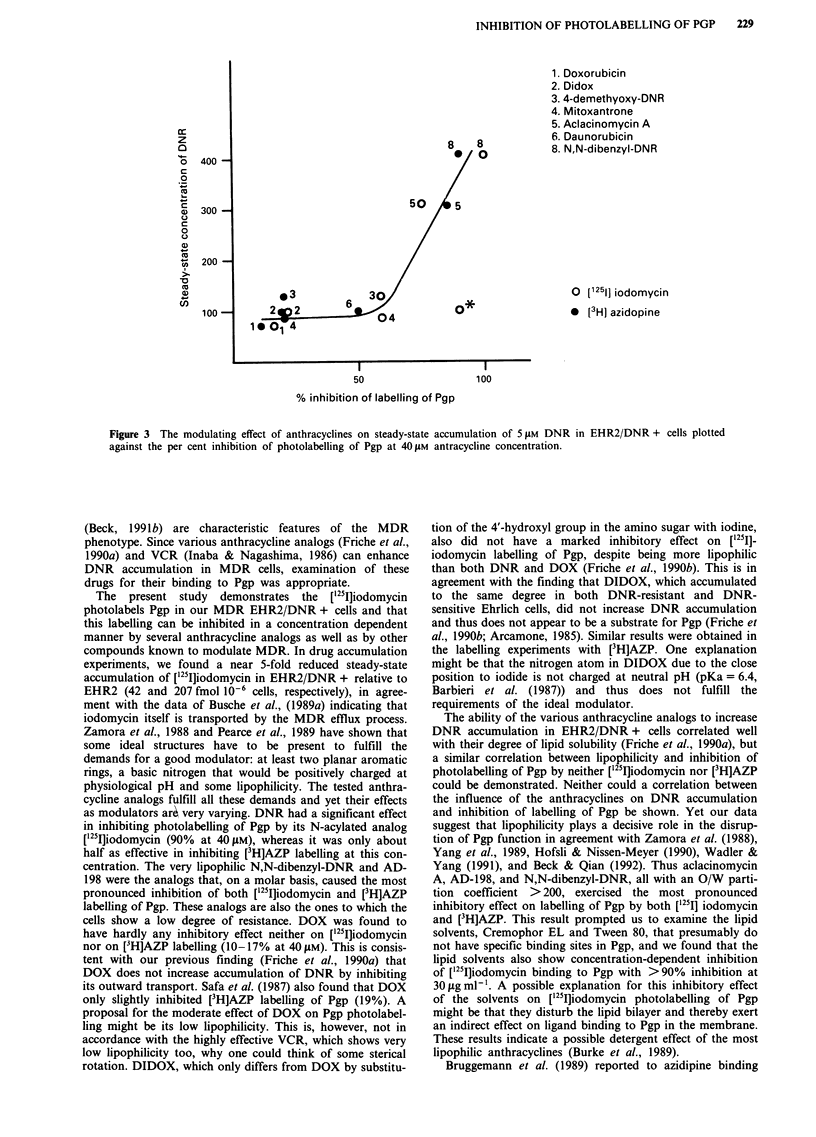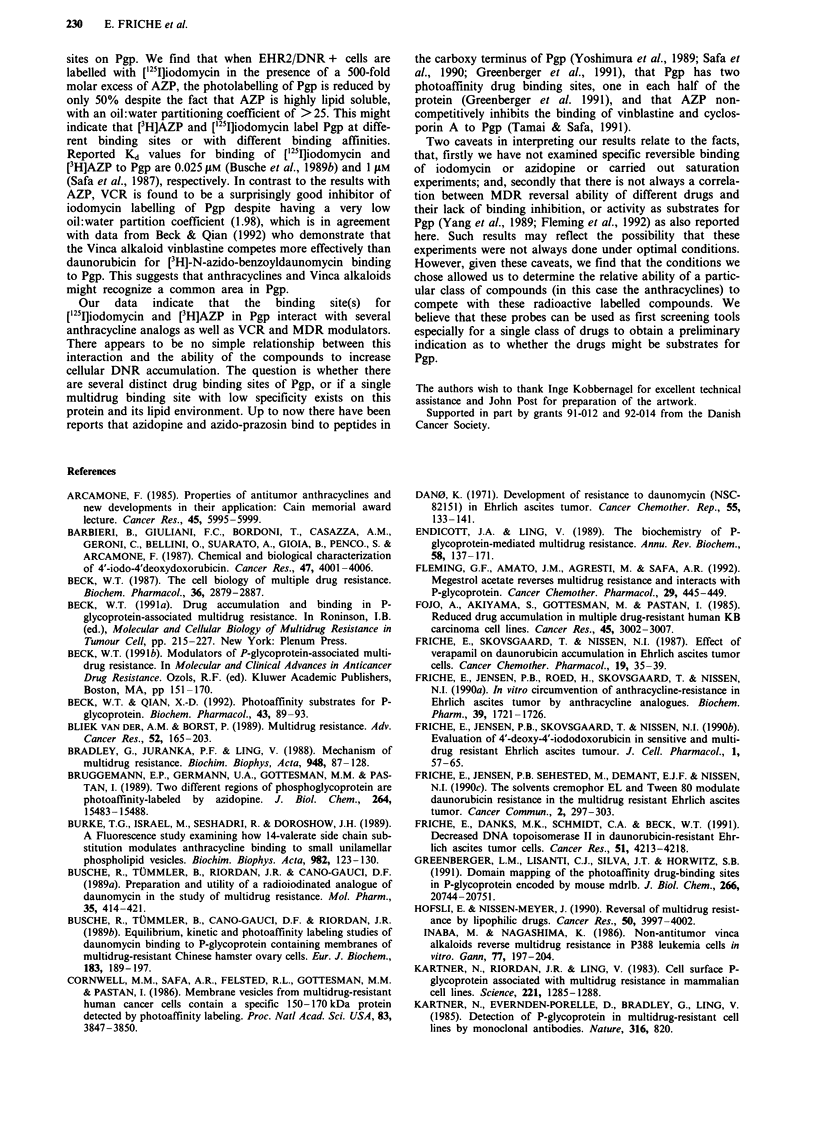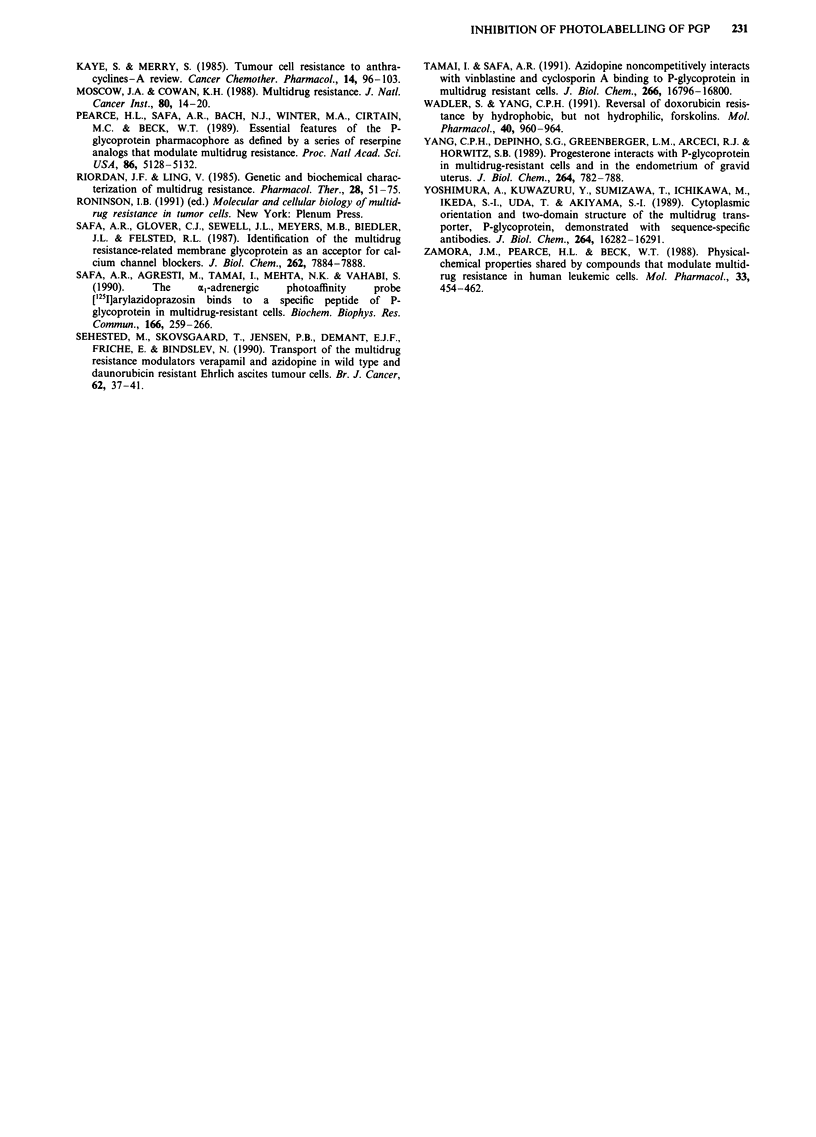# Effect of anthracycline analogs on photolabelling of p-glycoprotein by [125I]iodomycin and [3H]azidopine: relation to lipophilicity and inhibition of daunorubicin transport in multidrug resistant cells.

**DOI:** 10.1038/bjc.1993.44

**Published:** 1993-02

**Authors:** E. Friche, E. J. Demant, M. Sehested, N. I. Nissen

**Affiliations:** Department of Medicine and Hematology, Rigshospitalet-University Hospital, Copenhagen, Denmark.

## Abstract

**Images:**


Br. J. Cancer (1993), 67, 226–231 © Macmillan Press Ltd., 1993

### Effect of anthracycline analogs on photolabelling of p-glycoprotein by [$^{125}$I]iodomycin and [$^{3}$H]azidopine: Relation to lipophilicity and inhibition of daunorubicin transport in multidrug resistant cells

E. Friche[1]*, E.J.F. Demant[2], M. Sehested[3] & N.I. Nissen[1]

[1]*Department of Medicine and Hematology, L 4042, Rigshospitalet-University Hospital, 9, Blegdamsvej, DK-2100 Copenhagen;* [2]*Department of Biochemistry C, The Panum Institute, University of Copenhagen, DK-2200 Copenhagen;* [3]*Department of Pathology, Sundby Hospital, DK-2300 Copenhagen, Denmark.*

**Summary** Eight anthracycline analogs that have been shown to modulate multidrug resistance (Friche *et al.*, *Biochem. Pharmacol.*, **39**, 1721–1726; 1990) were tested for their inhibitory effect on the photolabelling of P-glycoprotein. We photoaffinity labelled P-glycoprotein in daunorubicin (DNR) resistant Ehrlich ascites tumour cells (EHR2/DNR + ) with a [$^{125}$I]iodinated Bolton-Hunter derivative of daunorubicin ([$^{125}$I]iodomycin) and with [$^{3}$H]azidopine. The photolabelling of P-glycoprotein by [$^{125}$I]iodomycin was inhibited more than 50% by 10 μM (1000-fold molar excess) of DNR (52%), N,N-dibenzyl-DNR (52%), and N-benzyladriamycin-14-valerate (AD-198) (85%). Vincristine at 10 μM inhibited [$^{125}$I]iodomycin labelling of P-glycoprotein by 95%. Thus vincristine was more potent than any of the eight anthracyclines tested, despite its relatively low lipophilicity. Increasing the concentration of DNR, AD-198 and N,N-dibenzyl-DNR to 40 μM resulted in 90, 99.5 and 99.5% inhibition of P-glycoprotein labelling by [$^{125}$I]iodomycin, respectively. In comparison with the other anthracycline analogs, N,N-dibenzyl-DNR and Ad-198 were also found to exert the greatest inhibition of [$^{3}$H]azidopine labelling of P-glycoprotein (about 90% at 100-fold molar excess). The solvents Cremophor EL and Tween 80 (30 μg ml$^{-1}$; 0.003% v/v), which are modulators of multidrug resistance in EHR2/DNR + cells, also inhibited [$^{125}$I]iodomycin labelling >90%. We showed earlier that there is a correlation between the lipid solubility within the anthracycline group of MDR-associated drugs and their ability to enhance DNR accumulation in EHR2/DNR + cells but a corresponding correlation to lipophilicity when it comes to the inhibitory effect on the specific photolabelling of Pgp ligand binding sites could not be demonstrated. Neither could a correlation between the modulating effect of the analogs on DNR accumulation and inhibition on the labelling of Pgp be demonstrated. With increasing lipophilicity of the analogs it seems that the chemical structure plays a lesser role, and the degree of lipophilicity becomes a more important feature.

Anthracyclines are among the most valuable cytostatic agents in clinical use. Their usefulness is, however, limited by the occurrence of tumour cells with the multidrug resistance (MDR) phenotype (Beck, 1987; Bliek & Borst, 1989; Endicott & Ling, 1989; Kaye & Merry, 1985; Moscow & Cowan, 1988). MDR cells have an enhanced amount of a 150–170 kDa plasma membrane glycoprotein, P-glycoprotein (Pgp) (Kartner *et al.*, 1983 and 1985; Cornwell *et al.*, 1986; Bradley *et al.*, 1988; Beck, 1991*a*; Roninson, 1991), that may function as a low-specificity transport protein to facilitate the outward transport of cytotoxic agents including the anthracyclines. Thus, increased drug efflux and therefore decreased accumulation is seen in MDR cells not only to the drug for which resistance is developed but also to a wide spectrum of chemically unrelated cytostatics (Bradley *et al.*, 1988; Fojo *et al.*, 1985). A variety of compounds have been shown to bind to Pgp: Vinca alkaloids, progesterone, azidopine, verapamil analogs, prazosin, iodomycin, and N-azido-benzoyldaunomycin (recently reviewed by Beck & Qian, 1992). We have reported earlier, that several anthracycline analogs *in vitro* were able to modulate DNR-resistance in Ehrlich ascites tumour cells by enhancing the drug accumulation (Friche *et al.*, 1990*a*). The aim of this study was to examine these analogs for their ability to compete for ligand binding to Pgp and thus service as inhibitors for the outward drug transport system in MDR cells. Anthracycline inter-actions with Pgp were assessed using the specific photolabelling of the protein with [$^{125}$I]iodomycin, a [$^{125}$I]iodinated Bolton-Hunter derivative of DNR (Busche *et al.*, 1989a) and with [$^{3}$H]azidopine ([$^{3}$H]AZP) which photolabels Pgp in plasma membrane vesicles from EHR2/DNR + cells (Sehested *et al.*, 1990) as well as in whole cells (Safa *et al.*, 1987; Friche *et al.*, 1990*c*). Further, we have studied the inhibitory effects of AZP, vincristine, and detergents on the photoreaction of these compounds with Pgp.

### Material and methods

### *Chemicals*

Duanorubicin (DNR); Adriamycin$^{R}$ (Doxorubicin/HCl, DOX); 4′-deoxy-4′-iododoxorubicin (DIDOX); and 4-demethoxy-DNR were kindly supplied from Farmitalia, Carlo Erba (Milan, Italy). N,N-dibenzyl-DNR was a gift from Dr Nicholas Bachur, (Baltimore, MD) and N-benzyladriamycin-14-valerate (AD 198) a gift from Professor M. Israel (Memphis, TN). Mitoxantrone was purchased from Lederle (New Jersey, USA), Aclacinomycin A from Lundbeck & Co A/S (Copenhagen, Denmark), Oncovin$^{R}$ (Vincristine/HCl, VCR) from Eli Lilly Deutschland GMBH (Giessen, Germany), Cremophor EL from BASF Aktiengesellschaft (Ludwigshafen, Germany), and Tween 80 from the Sigma Chemical Co. (St. Louis, MO). [$^{125}$I]-Bolton-Hunter reagent (2000 Ci mmol$^{-1}$), [$^{3}$H]AZP (42 Ci mmol$^{-1}$) and non-radioactive AZP were purchased from Amersham International (Amersham, UK). All other chemicals were of analytical grade. Organic solvents were distilled prior to use. Triethylamine was distilled over ninhydrin and anhydrous methanol was stored over molecular sieve (3 Å).

### *Tumour cells*

Cells were either the wild-type Ehrlich ascites tumor line (EHR2) or the corresponding DNR-resistant line (EHR2/DNR + ) (Danø, 1971) displaying both the MDR phenotype (Sehested *et al.*, 1990) and as recently shown, decreased

Correspondence: E. Friche.
Received 22 April 1992; and in revised form 21 September 1992.

amount of topoisomerase II (Friche *et al.*, 1991). Resistance to DNR was developed and maintained *in vivo* in mice as previously described in detail (Danø, 1971). *In vivo* the subline is at least 16-fold resistant to DNR (Friche *et al.*, 1987) and *in vitro* in a clonogenic assay about 80-fold resistant (Friche *et al.*, 1990*b*). No drug treatment was given to the resistant tumour during the last passage before an *in vitro* experiment.

### *Synthesis of [$^{125}$I]iodomycin*

A modification of the procedure of Busche *et al.* (1989*a*) was used for the synthesis of [$^{125}$I]iodomycin (Figure 1). DNR (80 nmol) in 100 µl of dry methanol containing 1 mM triethylamine was reacted with a solution of 0.5 nmol (1 mCi) monoiodinated Bolton-Hunter reagent (N-succinimidyl-3-(4-hydroxy, 5-([$^{125}$I]iodophenyl)propionate) in 200 µl of dry benzene containing 0.2% dimethylformamide. After 2 h at room temperature, the product was purified by TLC on precoated 0.25 mm silica gel 60 plates (Merck, Germany) with the solvent system ethyl acetate/diethyl ether (2:1, v/v, $R_f$ 0.12), and extracted from the silica by ethanol (2 × 1 ml). The solvent was evaporated under a stream of nitrogen to a final volume of about 200 µl. The radiochemical yields on the basis of Bolton-Hunter reagent were typically 50–60%.

### *Drug accumulation and lipophilicity*

Drug accumulation experiments with EHR2 and EHR2/DNR + cells and determination of drug lipophilicity by octanol/water partitioning were carried out as described previously (Friche *et al.*, 1990 *a,b,c*).

### *Photolabelling of Pgp by [$^{125}$I]iodomycin and [$^{3}$H]AZP*

[$^{125}$I]iodomycin and [$^{3}$H]AZP in ethanolic solutions were added at a final concentration of 10 nM and 0.4 µM, respectively (1% v/v final ethanol) to cell suspensions (2 × $10^6$ cells ml$^{-1}$) in phosphate buffer (Friche *et al.*, 1990*a*) containing 10 mM glucose. Aliquots (300 µl) were transferred to a Micro Well plate (Nunc, Roskilde, Denmark), and various concentrations of anthracyclines, or MDR modulators were added (see Tables I and II). The samples were incubated for 30 min at 37°C, before irradiation under 254 nm UV light for 20 min. The cells were then transferred to Eppendrof tubes and centrifuged for 2 min at 10,000 rpm. Fifty µl of 1% NP 40 were added to dissolve the cell pellets and after 10 min, the suspensions were centrifuged for 5 min at 15,000 rpm. The resulting supernatants were transferred to new tubes and 10 µl of SDS (10%) and 10 µl of bromphenolblue in 50% glycerol were added before analysis by SDS PAGE using 10% gels. Control experiments confirmed complete extraction of Pgp from the cells by this procedure. Following electrophoresis, the gels were stained for protein with Coomassie Brilliant blue. In experiments with [$^{3}$H]AZP, the gels were prepared for fluorography in Amplify™ (Amersham International, UK). Fluorographs were obtained with Kodak X-Omat film (Eastman Kodak Co., Rochester, NY) exposed for 4 days at − 80°C. Quantitation of [$^{125}$I]iodomycin and [$^{3}$H]AZP labelling of Pgp was carried out by photodensitometry of the fluorographs using a LKB UltraScan Laser Densitometer (Sweden).

**Table II** Per cent inhibition of [$^{125}$I]iodomycin photolabelling by Pgp by various MDR modulators

| | *0* | *1 µM* | *5 µM* | *10 µM* | *O/W* |
|---|---|---|---|---|---|
| Vincristine | 0 | 53.1 | 78.6 | 94.3 | 1.98 |
| | *0* | *1 µM* | *5 µM* | *10 µM* | *O/W* |
| Azidopine | 0 | 6.7 | 50.0 | 67.9 | 27.61 |
| | *0* | *1 µg ml$^{-1}$* | *10 µg ml$^{-1}$* | *30 µg ml$^{-1}$* | |
| Cremophor EL | 0 | 28.6 | 71.5 | 92.8 | |
| Tween 80 | 0 | 73.4 | 91.2 | 97.7 | |

Experimental details as in Table I, but with varying concentrations of modulators as indicated. Results are expressed as per cent of the control without added modulator. The number of experiments varies from 2–5 for the different drugs with a variation ~ 10%.

### Results

### *Inhibitory effect of DNR, DOX and N,N-dibenzyl-DNR on [$^{125}$I]iodomycin photolabelling of Pgp*

Photolabelling of proteins in intact EHR2 and Pgp-MDR EHR2/DNR + cells with [$^{125}$I]iodomycin and [$^{3}$H]AZP and the effects of DNR, DOX and N,N-dibenzyl-DNR on this labelling are shown in Figure 2. In EHR2/DNR + cells, a distinct $M_r$ 170,000 band at the position of Pgp was labelled with 10 nM [$^{125}$I]iodomycin (lane 2) and with 0.4 µM [$^{3}$H]-AZP (lane 12). No labelling in this region was seen in the EHR2 cells (lane 1). The specific labelling of Pgp by [$^{125}$I]iodomycin was inhibited in a dose-dependent manner by the anthracyclines, when they were preincubated with the cells for 30 min, at which time steady-state accumulation was reached for all the anthracyclines. Ten µM (1000-fold molar excess) DNR or N,N-dibenzyl-DNR inhibited labelling by about 50% (lanes 3 and 9), while DOX at this concentration did not cause significant inhibition of labelling (lane 6). At 20 µM, 83%, 0%, and 97% inhibition was observed by DNR, DOX, and N,N-dibenzyl-DNR, respectively (lanes 10, 7 and 4). A further increase in DNR and N,N-dibenzyl-DNR concentration to 40 µM resulted in a nearly total inhibition of

**Table I** Percent inhibitory effect of anthracyclines on [$^{125}$I]iodomycin and ([$^{3}$H]AZP) photolabelling of Pgp in EHR2/DNR + cells and their lipid solubility

| *Drug* | *10 µM* | *20 µM* | *40 µM* | *O/W* |
|---|---|---|---|---|
| Doxorubicin | 0 (0)[a] | 0 (10.5) | 16.8 (11.0) | 1.22 |
| DIDOX | 20.6 (0) | 17.5 (0) | 22.5 (19.7) | 14.51 |
| 4-demethoxy-daunorubicin | 35.9 ( 1.5) | 52.2 ( 3.6) | 58.3 (19.8) | 13.08 |
| Mitoxantrone | 42.9 ( 7.5) | 56.9 (15.6) | 58.8 (19.5) | 5.06 |
| Aclacinomycin A | 22.6 (65.3) | 52.0 (74.7) | 75.3 (85.7) | >200 |
| Daunorubicin | 52.5 ( 8.0) | 82.7 (33.2) | 90.6 (50.0) | 7.85 |
| AD-198 | 85.0 (74.1) | 95.8 (81.7) | >99 (90.9) | >200 |
| N,N,-dibenzyl-daunorubicin | 52.4 (75.2) | 97.3 (87.1) | >99 (90.5) | >200 |

[a]Numbers in parentheses represent inhibition of [$^{3}$H]AZP binding. Numbers not in parentheses represent inhibition of [$^{125}$I]iodomycin binding.
Suspensions of EHR2/DNR + cells (2 × $10^6$ cells ml$^{-1}$ were incubated for 30 min at 37°C with either iodomycin (10 nM) or [$^{3}$H]AZP (0.4 µM) in the absence (control) and in the presence of various concentrations of anthracyclines (10–40 µM). See 'Materials and methods' for details. Lipid solubility (O/W) of the anthracyclines was determined by measuring the partition of the drugs between an aqueous phase and octanol as described in Friche *et al.* (1990*a*). Part of the lipid solubility data is published in Friche *et al.* (1990*a*). Results are expressed as per cent of the control without added anthracycline.

labelling (90 and >99% respectively) (lanes 11 and 5). Still, DOX had little inhibitory effect (17%, lane 8).

*Relationship between anthracycline lipophilicity and inhibition of Pgp photolabelling*

Table I lists the anthracycline analogs and shows their inhibitory effect on [$^{125}$I]iodomycin and [$^{3}$H]AZP (numbers in brackets) labelling of Pgp. Included in Table I is also the lipid solubility of these analogs measured as the octanol/water partition coefficient at pH 7.45. It is seen that no simple relationship between lipophilicity of the anthracycline analogs and their capacity to inhibit the photolabelling of Pgp could be demonstrated.

*Relationship between modulating effect of anthracyclines on DNR accumulation and inhibition of Pgp photolabelling*

We have shown earlier that some anthracyclines were modulators of DNR resistance (Friche *et al.*, 1990*a*). Therefore a possible coherence between modulating effect of the anthracyclines on DNR accumulation and inhibition of Pgp photolabelling was examined. A correlation between the influence of the analogs on steady-state accumulation of DNR and their inhibitory effect on labelling of Pgp by [$^{125}$I]iodomycin or [$^{3}$H]AZP could not be demonstrated (Figure 3). Nonetheless, the two highly lipophilic analogs, Aclacinomycin A and N,N-dibenzyl-DNR, which were the most effective to inhibit labelling of Pgp also did increase accumulation of DNR the most at equimolar concentrations (= 5 μM).

*Inhibitory effect of VCR, AZP and detergents on [$^{125}$I]iodomycin photolabelling of Pgp*

We also examined the ability of other compounds to inhibit [$^{125}$I]iodomycin labelling of Pgp (Table II). VCR (1 μM), to

**Figure 1** Structure of [$^{125}$I]iodomycin.

which EHR2/DNR + cells are cross-resistant, inhibited the labelling by about 50%, and 10 μM VCR almost completely blocked this labelling. AZP hardly inhibited [$^{125}$I]iodomycin labelling of Pgp at 1 μM, while 5 μM resulted in a 50% inhibition. Finally, the detergents Cremophor EL and Tween 80, both of which have been shown to reverse drug-resistance in EHR2/DNR + cells at 30 μg ml$^{-1}$ (0.003% v/v) (Friche *et al.*, 1990*c*), also inhibited photolabelling of Pgp by [$^{125}$I]iodomycin by >90% when present in the reaction mixture at this concentration.

### Discussion

Overexpression of Pgp (Riordan & Ling, 1985) and inhibition of its function by several classes of membrane-active drugs

**Figure 2** Effects of DNR, DOX and N,N-dibenzyl-DNR on [$^{125}$I]iodomycin photolabelling of Pgp in EHR2/DNR + cells. EHR2/DNR + cells (2 × 10$^{6}$ cells/lane) were labelled with [$^{125}$I]iodomycin (10 nM) and separation of total cell protein was by SDS-PAGE as described in 'Materials and methods'. The position of Pgp in the gel is indicated as 170 kDa. Photolabelling was carried out in the absence of drugs (lane 2) and in the presence of 10, 20 or 40 μM of either DNR (lanes 9–11), DOX (lanes 6–8), or N,N-dibenzyl-DNR (lanes 3–5) preincubated with the cells for 30 min. Lane 1 shows EHR2 as negative control, and lane 12, EHR2/DNR + cells photolabelled with [$^{3}$H]AZP (0.4 μM). The fluorogram was developed with a film exposure of 4 days. The experiment was done three times, and a representative gel is shown.

**Figure 3** The modulating effect of anthracyclines on steady-state accumulation of 5 μM DNR in EHR2/DNR + cells plotted against the per cent inhibition of photolabelling of Pgp at 40 μM antracycline concentration.

(Beck, 1991*b*) are characteristic features of the MDR phenotype. Since various anthracycline analogs (Friche *et al.*, 1990*a*) and VCR (Inaba & Nagashima, 1986) can enhance DNR accumulation in MDR cells, examination of these drugs for their binding to Pgp was appropriate.

The present study demonstrates the [$^{125}$I]iodomycin photolabels Pgp in our MDR EHR2/DNR + cells and that this labelling can be inhibited in a concentration dependent manner by several anthracycline analogs as well as by other compounds known to modulate MDR. In drug accumulation experiments, we found a near 5-fold reduced steady-state accumulation of [$^{125}$I]iodomycin in EHR2/DNR + relative to EHR2 (42 and 207 fmol $10^{-6}$ cells, respectively), in agreement with the data of Busche *et al.*, (1989*a*) indicating that iodomycin itself is transported by the MDR efflux process. Zamora *et al.*, 1988 and Pearce *et al.*, 1989 have shown that some ideal structures have to be present to fulfill the demands for a good modulator: at least two planar aromatic rings, a basic nitrogen that would be positively charged at physiological pH and some lipophilicity. The tested anthracycline analogs fulfill all these demands and yet their effects as modulators are very varying. DNR had a significant effect in inhibiting photolabelling of Pgp by its N-acylated analog [$^{125}$I]iodomycin (90% at 40 μM), whereas it was only about half as effective in inhibiting [$^{3}$H]AZP labelling at this concentration. The very lipophilic N,N-dibenzyl-DNR and AD-198 were the analogs that, on a molar basis, caused the most pronounced inhibition of both [$^{125}$I]iodomycin and [$^{3}$H]AZP labelling of Pgp. These analogs are also the ones to which the cells show a low degree of resistance. DOX was found to have hardly any inhibitory effect neither on [$^{125}$I]iodomycin nor on [$^{3}$H]AZP labelling (10–17% at 40 μM). This is consistent with our previous finding (Friche *et al.*, 1990*a*) that DOX does not increase accumulation of DNR by inhibiting its outward transport. Safa *et al.* (1987) also found that DOX only slightly inhibited [$^{3}$H]AZP labelling of Pgp (19%). A proposal for the moderate effect of DOX on Pgp photolabelling might be its low lipophilicity. This is, however, not in accordance with the highly effective VCR, which shows very low lipophilicity too, why one could think of some sterical rotation. DIDOX, which only differs from DOX by substitution of the 4′-hydroxyl group in the amino sugar with iodine, also did not have a marked inhibitory effect on [$^{125}$I]-iodomycin labelling of Pgp, despite being more lipophilic than both DNR and DOX (Friche *et al.*, 1990*b*). This is in agreement with the finding that DIDOX, which accumulated to the same degree in both DNR-resistant and DNR-sensitive Ehrlich cells, did not increase DNR accumulation and thus does not appear to be a substrate for Pgp (Friche *et al.*, 1990*b*; Arcamone, 1985). Similar results were obtained in the labelling experiments with [$^{3}$H]AZP. One explanation might be that the nitrogen atom in DIDOX due to the close position to iodide is not charged at neutral pH (pKa = 6.4, Barbieri *et al.* (1987)) and thus does not fulfill the requirements of the ideal modulator.

The ability of the various anthracycline analogs to increase DNR accumulation in EHR2/DNR + cells correlated well with their degree of lipid solubility (Friche *et al.*, 1990*a*), but a similar correlation between lipophilicity and inhibition of photolabelling of Pgp by neither [$^{125}$I]iodomycin nor [$^{3}$H]AZP could be demonstrated. Neither could a correlation between the influence of the anthracyclines on DNR accumulation and inhibition of labelling of Pgp be shown. Yet our data suggest that lipophilicity plays a decisive role in the disruption of Pgp function in agreement with Zamora *et al.* (1988), Yang *et al.*, 1989, Hofsli & Nissen-Meyer (1990), Wadler & Yang (1991), and Beck & Qian (1992). Thus aclacinomycin A, AD-198, and N,N-dibenzyl-DNR, all with an O/W partition coefficient >200, exercised the most pronounced inhibitory effect on labelling of Pgp by both [$^{125}$I] iodomycin and [$^{3}$H]AZP. This result prompted us to examine the lipid solvents, Cremophor EL and Tween 80, that presumably do not have specific binding sites in Pgp, and we found that the lipid solvents also show concentration-dependent inhibition of [$^{125}$I]iodomycin binding to Pgp with >90% inhibition at 30 μg ml$^{-1}$. A possible explanation for this inhibitory effect of the solvents on [$^{125}$I]iodomycin photolabelling of Pgp might be that they disturb the lipid bilayer and thereby exert an indirect effect on ligand binding to Pgp in the membrane. These results indicate a possible detergent effect of the most lipophilic anthracyclines (Burke *et al.*, 1989).

Bruggemann *et al.* (1989) reported to azidipine binding sites on Pgp. We find that when EHR2/DNR + cells are labelled with [$^{125}$I]iodomycin in the presence of a 500-fold molar excess of AZP, the photolabelling of Pgp is reduced by only 50% despite the fact that AZP is highly lipid soluble, with an oil:water partitioning coefficient of >25. This might indicate that [$^{3}$H]AZP and [$^{125}$I]iodomycin label Pgp at different binding sites or with different binding affinities. Reported $K_d$ values for binding of [$^{125}$I]iodomycin and [$^{3}$H]AZP to Pgp are 0.025 μM (Busche *et al.*, 1989*b*) and 1 μM (Safa *et al.*, 1987), respectively. In contrast to the results with AZP, VCR is found to be a surprisingly good inhibitor of iodomycin labelling of Pgp despite having a very low oil:water partition coefficient (1.98), which is in agreement with data from Beck & Qian (1992) who demonstrate that the Vinca alkaloid vinblastine competes more effectively than daunorubicin for [$^{3}$H]-N-azido-benzoyldaunomycin binding to Pgp. This suggests that anthracyclines and Vinca alkaloids might recognize a common area in Pgp.

Our data indicate that the binding site(s) for [$^{125}$I]iodomycin and [$^{3}$H]AZP in Pgp interact with several anthracycline analogs as well as VCR and MDR modulators. There appears to be no simple relationship between this interaction and the ability of the compounds to increase cellular DNR accumulation. The question is whether there are several distinct drug binding sites of Pgp, or if a single multidrug binding site with low specificity exists on this protein and its lipid environment. Up to now there have been reports that azidopine and azido-prazosin bind to peptides in the carboxy terminus of Pgp (Yoshimura *et al.*, 1989; Safa *et al.*, 1990; Greenberger *et al.*, 1991), that Pgp has two photoaffinity drug binding sites, one in each half of the protein (Greenberger *et al.* 1991), and that AZP non-competitively inhibits the binding of vinblastine and cyclosporin A to Pgp (Tamai & Safa, 1991).

Two caveats in interpreting our results relate to the facts, that, firstly we have not examined specific reversible binding of iodomycin or azidopine or carried out saturation experiments; and, secondly that there is not always a correlation between MDR reversal ability of different drugs and their lack of binding inhibition, or activity as substrates for Pgp (Yang *et al.*, 1989; Fleming *et al.*, 1992) as also reported here. Such results may reflect the possibility that these experiments were not always done under optimal conditions. However, given these caveats, we find that the conditions we chose allowed us to determine the relative ability of a particular class of compounds (in this case the anthracyclines) to compete with these radioactive labelled compounds. We believe that these probes can be used as first screening tools especially for a single class of drugs to obtain a preliminary indication as to whether the drugs might be substrates for Pgp.

The authors wish to thank Inge Kobbernagel for excellent technical assistance and John Post for preparation of the artwork.

Supported in part by grants 91-012 and 92-014 from the Danish Cancer Society.

### References

ARCAMONE, F. (1985). Properties of antitumor anthracyclines and new developments in their application: Cain memorial award lecture. *Cancer Res.*, **45**, 5995–5999.

BARBIERI, B., GIULIANI, F.C., BORDONI, T., CASAZZA, A.M., GERONI, C., BELLINI, O., SUARATO, A., GIOIA, B., PENCO., S. & ARCAMONE, F. (1987). Chemical and biological characterization of 4'-iodo-4'deoxydoxorubicin. *Cancer Res.*, **47**, 4001–4006.

BECK, W.T. (1987). The cell biology of multiple drug resistance. *Biochem. Pharmacol.*, **36**, 2879–2887.

BECK, W.T. (1991*a*). Drug accumulation and binding in P-glycoprotein-associated multidrug resistance. In Roninson, I.B. (ed.), *Molecular and Cellular Biology of Multidrug Resistance in Tumour Cell*, pp. 215–227. New York: Plenum Press.

BECK, W.T. (1991*b*). Modulators of *P*-glycoprotein-associated multidrug resistance. In *Molecular and Clinical Advances in Anticancer Drug Resistance*. Ozols, R.F. (ed). Kluwer Academic Publishers, Boston, MA, pp 151–170.

BECK, W.T. & QIAN, X.-D. (1992). Photoaffinity substrates for P-glycoprotein. *Biochem. Pharmacol.*, **43**, 89–93.

BLIEK VAN DER, A.M. & BORST, P. (1989). Multidrug resistance. *Adv. Cancer Res.*, **52**, 165–203.

BRADLEY, G., JURANKA, P.F. & LING, V. (1988). Mechanism of multidrug resistance. *Biochim. Biophys, Acta*, **948**, 87–128.

BRUGGEMANN, E.P., GERMANN, U.A., GOTTESMAN, M.M. & PASTAN, I. (1989). Two different regions of phosphoglycoprotein are photoaffinity-labeled by azidopine. *J. Biol. Chem.*, **264**, 15483–15488.

BURKE, T.G., ISRAEL, M., SESHADRI, R. & DOROSHOW, J.H. (1989). A Fluorescence study examining how 14-valerate side chain substitution modulates anthracycline binding to small unilamellar phospholipid vesicles. *Biochim. Biophys. Acta*, **982**, 123–130.

BUSCHE, R., TÜMMLER, B., RIORDAN, J.R. & CANO-GAUCI, D.F. (1989*a*). Preparation and utility of a radioiodinated analogue of daunomycin in the study of multidrug resistance. *Mol. Pharm.*, **35**, 414–421.

BUSCHE, R., TÜMMLER, B., CANO-GAUCI, D.F. & RIORDAN, J.R. (1989*b*). Equilibrium, kinetic and photoaffinity labeling studies of daunomycin binding to P-glycoprotein containing membranes of multidrug-resistant Chinese hamster ovary cells. *Eur. J. Biochem.*, **183**, 189–197.

CORNWELL, M.M., SAFA, A.R., FELSTED, R.L., GOTTESMAN, M.M. & PASTAN, I. (1986). Membrane vesicles from multidrug-resistant human cancer cells contain a specific 150–170 kDa protein detected by photoaffinity labeling. *Proc. Natl Acad. Sci. USA*, **83**, 3847–3850.

DANØ, K. (1971). Development of resistance to daunomycin (NSC-82151) in Ehrlich ascites tumor. *Cancer Chemother. Rep.*, **55**, 133–141.

ENDICOTT, J.A. & LING, V. (1989). The biochemistry of P-glycoprotein-mediated multidrug resistance. *Annu. Rev. Biochem.*, **58**, 137–171.

FLEMING, G.F., AMATO, J.M., AGRESTI, M. & SAFA, A.R. (1992). Megestrol acetate reverses multidrug resistance and interacts with P-glycoprotein. *Cancer Chemother. Pharmacol.*, **29**, 445–449.

FOJO, A., AKIYAMA, S., GOTTESMAN, M. & PASTAN, I. (1985). Reduced drug accumulation in multiple drug-resistant human KB carcinoma cell lines. *Cancer Res.*, **45**, 3002–3007.

FRICHE, E., SKOVSGAARD, T. & NISSEN, N.I. (1987). Effect of verapamil on daunorubicin accumulation in Ehrlich ascites tumor cells. *Cancer Chemother. Pharmacol.*, **19**, 35–39.

FRICHE, E., JENSEN, P.B., ROED, H., SKOVSGAARD, T. & NISSEN, N.I. (1990*a*). *In vitro* circumvention of anthracycline-resistance in Ehrlich ascites tumor by anthracycline analogues. *Biochem. Pharm.*, **39**, 1721–1726.

FRICHE, E., JENSEN, P.B., SKOVSGAARD, T. & NISSEN, N.I. (1990*b*). Evaluation of 4'-deoxy-4'-iododoxorubicin in sensitive and multidrug resistant Ehrlich ascites tumour. *J. Cell. Pharmacol.*, **1**, 57–65.

FRICHE, E., JENSEN, P.B. SEHESTED, M., DEMANT, E.J.F. & NISSEN, N.I. (1990*c*). The solvents cremophor EL and Tween 80 modulate daunorubicin resistance in the multidrug resistant Ehrlich ascites tumor. *Cancer Commun.*, **2**, 297–303.

FRICHE, E., DANKS, M.K., SCHMIDT, C.A. & BECK, W.T. (1991). Decreased DNA topoisomerase II in daunorubicin-resistant Ehrlich ascites tumor cells. *Cancer Res.*, **51**, 4213–4218.

GREENBERGER, L.M., LISANTI, C.J., SILVA, J.T. & HORWITZ, S.B. (1991). Domain mapping of the photoaffinity drug-binding sites in P-glycoprotein encoded by mouse mdrlb. *J. Biol. Chem.*, **266**, 20744–20751.

HOFSLI, E. & NISSEN-MEYER, J. (1990). Reversal of multidrug resistance by lipophilic drugs. *Cancer Res.*, **50**, 3997–4002.

INABA, M. & NAGASHIMA, K. (1986). Non-antitumor vinca alkaloids reverse multidrug resistance in P388 leukemia cells *in vitro*. *Gann*, **77**, 197–204.

KARTNER, N., RIORDAN, J.R. & LING, V. (1983). Cell surface P-glycoprotein associated with multidrug resistance in mammalian cell lines. *Science*, **221**, 1285–1288.

KARTNER, N., EVERNDEN-PORELLE, D., BRADLEY, G., LING, V. (1985). Detection of P-glycoprotein in multidrug-resistant cell lines by monoclonal antibodies. *Nature*, **316**, 820.

KAYE, S. & MERRY, S. (1985). Tumour cell resistance to anthracyclines–A review. *Cancer Chemother. Pharmacol.*, **14**, 96–103.

MOSCOW, J.A. & COWAN, K.H. (1988). Multidrug resistance. *J. Natl. Cancer Inst.*, **80**, 14–20.

PEARCE, H.L., SAFA, A.R., BACH, N.J., WINTER, M.A., CIRTAIN, M.C. & BECK, W.T. (1989). Essential features of the P-glycoprotein pharmacophore as defined by a series of reserpine analogs that modulate multidrug resistance. *Proc. Natl Acad. Sci. USA*, **86**, 5128–5132.

RIORDAN, J.F. & LING, V. (1985). Genetic and biochemical characterization of multidrug resistance. *Pharmacol. Ther.*, **28**, 51–75.

RONINSON, I.B. (1991) (ed.) *Molecular and cellular biology of multidrug resistance in tumor cells.* New York: Plenum Press.

SAFA, A.R., GLOVER, C.J., SEWELL, J.L., MEYERS, M.B., BIEDLER, J.L. & FELSTED, R.L. (1987). Identification of the multidrug resistance-related membrane glycoprotein as an acceptor for calcium channel blockers. *J. Biol. Chem.*, **262**, 7884–7888.

SAFA, A.R., AGRESTI, M., TAMAI, I., MEHTA, N.K. & VAHABI, S. (1990). The $\alpha_1$-adrenergic photoaffinity probe [$^{125}$I]arylazidoprazosin binds to a specific peptide of P-glycoprotein in multidrug-resistant cells. *Biochem. Biophys. Res. Commun.*, **166**, 259–266.

SEHESTED, M., SKOVSGAARD, T., JENSEN, P.B., DEMANT, E.J.F., FRICHE, E. & BINDSLEV, N. (1990). Transport of the multidrug resistance modulators verapamil and azidopine in wild type and daunorubicin resistant Ehrlich ascites tumour cells. *Br. J. Cancer*, **62**, 37–41.

TAMAI, I. & SAFA, A.R. (1991). Azidopine noncompetitively interacts with vinblastine and cyclosporin A binding to P-glycoprotein in multidrug resistant cells. *J. Biol. Chem.*, **266**, 16796–16800.

WADLER, S. & YANG, C.P.H. (1991). Reversal of doxorubicin resistance by hydrophobic, but not hydrophilic, forskolins. *Mol. Pharmacol.*, **40**, 960–964.

YANG, C.P.H., DEPINHO, S.G., GREENBERGER, L.M., ARCECI, R.J. & HORWITZ, S.B. (1989). Progesterone interacts with P-glycoprotein in multidrug-resistant cells and in the endometrium of gravid uterus. *J. Biol. Chem.*, **264**, 782–788.

YOSHIMURA, A., KUWAZURU, Y., SUMIZAWA, T., ICHIKAWA, M., IKEDA, S.-I., UDA, T. & AKIYAMA, S.-I. (1989). Cytoplasmic orientation and two-domain structure of the multidrug transporter, P-glycoprotein, demonstrated with sequence-specific antibodies. *J. Biol. Chem.*, **264**, 16282–16291.

ZAMORA, J.M., PEARCE, H.L. & BECK, W.T. (1988). Physical-chemical properties shared by compounds that modulate multidrug resistance in human leukemic cells. *Mol. Pharmacol.*, **33**, 454–462.